# Atoh8 in Development and Disease

**DOI:** 10.3390/biology11010136

**Published:** 2022-01-14

**Authors:** Satya Srirama Karthik Divvela, Darius Saberi, Beate Brand-Saberi

**Affiliations:** 1 Department of Anatomy and Molecular Embryology, Medical Faculty, Ruhr University Bochum, 44801 Bochum, Germany; Satya.Divvela@ruhr-uni-bochum.de; 2 Department of Neurology, University Medical Center, 37099 Göttingen, Germany; dariusleonard.saberikakhki@med.uni-goettingen.de

**Keywords:** Atoh8, Math6, development, cancer, BMP, iron-metabolism, homeostasis

## Abstract

**Simple Summary:**

bHLH transcription factors control a variety of functions during development and disease. Atoh8 is one such bHLH factor with unique characteristics in the ‘atonal’ family. Consistent with the other homologs of its family, Atoh8 was identified to regulate cell fate and differentiation during development. Furthermore, it was also found to regulate other mechanisms in adult and mature organisms such as cancer, iron homeostasis and stress response. This review systematically outlines the role of Atoh8 in development and pathologies. At the same time, this review also anticipates its potential involvement in basic cellular mechanisms such as proliferation, metabolism, apoptosis and autophagy.

**Abstract:**

Atoh8 belongs to a large superfamily of transcriptional regulators called basic helix-loop-helix (bHLH) proteins. bHLH proteins have been identified in a wide range of organisms from yeast to humans. The members of this special group of transcription factors were found to be involved not only in embryonic development but also in disease initiation and its progression. Given their importance in several fundamental processes, the translation, subcellular location and turnover of bHLH proteins is tightly regulated. Alterations in the expression of bHLH proteins have been associated with multiple diseases also in context with Atoh8 which seems to unfold its functions as both transcriptional activator and repressor. Like many other bHLH transcription factors, so far, Atoh8 has also been observed to be involved in both embryonic development and carcinogenesis where it mainly acts as tumor suppressor. This review summarizes our current understanding of Atoh8 structure, function and regulation and its complex and partially controversial involvement in development and disease.

## 1. Introduction

The basic helix-loop-helix (bHLH) proteins are an evolutionarily ancient superfamily of transcription factors that control a wide range of functions from proliferation to differentiation of tissues. Members of the bHLH family possess two highly conserved and functionally distinct domains namely ‘basic’ and ‘Helix-Loop-Helix’. The ‘basic’ domain enables the binding of these transcription factors to the major groove of DNA at the consensus sequences such as E-Boxes or N-Boxes. The ‘HLH’ domain aids in the interaction with other proteins either to form homodimers or heterodimers [1]. Based on the phylogenetic analysis and criteria such as consensus sequence binding, motif conservation, presence or absence of domains, the bHLH transcription factors are classified into six different groups named A, B, C, D, E and F [2,3].

bHLH transcription factors have been identified to be integral to multiple developmental processes. However, studying them broadly shows distinct expression patterns. The proteins that are expressed tissue- and cell-specifically and the proteins that are ubiquitously expressed. Concerning tissue-specific transcription factors, to date, several bHLH transcription factors have been identified to be essential for organogenesis such as in neurogenesis (Mash1, Math1 and NeuroD, Neurogenin), myogenesis (MyoD, Myogenin, Myf5 and Mrf4) and cardiac development (Hand1 and Hand2) [4,5,6]. These transcription factors are involved in the determination of cellular fate and their subsequent tissue-specific differentiation during development. However, on the other hand, the proteins that are ubiquitously expressed such as Myc, Mad, Max are involved in the regulation of cellular homeostasis, cell cycle and apoptosis [7]. Overall, based on the diversity in the dimerization of bHLH proteins and given the heterogeneity in recognizing the consensus sequences, this superfamily of proteins possesses the ability to control diverse functions by regulating and modulating the transcription of a wide range of target genes [8].

## 2. Atoh8

Atoh8 belongs to Group A of bHLH transcription factors that bind to the core consensus DNA sequence ‘CACCTG’ or ‘CAGCTG’ called E-Box. It is a member of the ‘Net’ family within the ‘Atonal’ superfamily [9,10]. Atoh8 was first identified in the context of neurogenesis where it was identified as another pro-neural transcription factor, like its homologs [11]. However, subsequent studies performed on zebrafish, chicken and mice have proved that Atoh8 is expressed ubiquitously in a temporally restricted way during embryonic development and during organogenesis.

**Figure 1:**
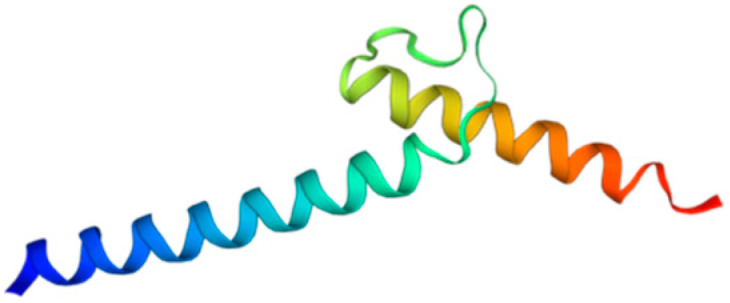
Structure of bHLH domain of Atoh8 predicted by Swiss-Model [14].

**Figure 2:**
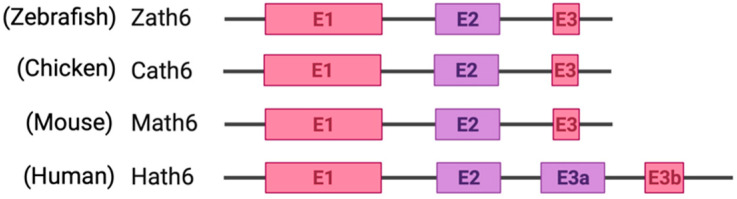
Schematic representation of exons and introns of Atoh8 in different orthologues. Zebrafish atonal homolog 6 (Zath6); Chicken atonal homolog 6 (Cath6); Mouse atonal homolog (Math6); Human atonal homolog 6 (Hath6).

Among metazoans, Atoh8 has undergone high sequence diversification making it difficult to predict its likely functions. Yet, the bHLH domain was observed to have remained highly conserved (Figure 1). As opposed to other members of the atonal superfamily, which are encoded by a single exon, Atoh8 is exceptionally encoded by three exons (exon1, exon2 and exon3b) from zebrafish to mammals (Figure 2). In humans, Chen and his colleagues identified ‘exon3a’ in addition to exons 1, 2 and 3b (Figure 2). In addition to this, a loop acceptor and donor close to exon3a and exon3b was further identified suggesting the possibility of alternative splicing in the case of Atoh8 [12]. A recent study performed to investigate the role of Atoh8 in breast cancer has not only identified this novel splice variant but also attributed it to have a discrete function compared to that of the originally identified Atoh8 [13].

Concerning regulatory aspects of Atoh8, the comparison of sequences upstream to Atoh8 in zebrafish, chicken, mice and humans revealed that the Atoh8 promoter has evolved from a ‘TATA box’ with a single transcriptional initiation site to the much more flexible ‘CpG islands’ with multiple transcriptional start sites that assist in fine-tuning of gene expression. This evolution in the promoter sequence also implies that Atoh8 has evolved from performing a specific function in zebrafish and chicken to multiple other functions in mice and humans, which is also in line with current literature where Atoh8 was shown to be ubiquitously expressed in mice during development [12,15]. Concerning the Atoh8 protein, in addition to the highly conserved bHLH domain, Atoh8 also possesses a proline-rich domain and serine-rich domain which are located in the N-terminus. The proline-rich domain in proteins such as p53 and Hex was implicated to repress transcription directly or by acting as co-repressors [16,17]. Although the proline-rich region of Atoh8 was attributed to its negative regulatory function, conclusive evidence of such repressive activity by the proline-rich domain is still missing. The serine-rich domain is liable for phosphorylation and dephosphorylation within proteins. The phosphorylation state of serine-rich domains is perceived to modulate gene expression temporally in the case of viral genes [18]. Likewise, the serine-rich domain of Atoh8 was identified to interact with calcineurin which is downstream of calcium signaling. The serine/threonine phosphatase Calcineurin was proposed to dephosphorylate Atoh8 thereby promoting its translocation into the nucleus where it exerts its function as a transcription factor [19].

### 2.1. Atoh8 in Development

#### 2.1.1. Atoh8 in Early Embryonic Development

Atoh8 can be detected very early during mouse embryonic development. Its expression was identified in the inner cell mass of the blastocyst (E3.5) where the cells are pluripotent. In addition to this, the dynamics in its localization was also reported, where the presence of Atoh8 protein was detected in the cytoplasm of the pluripotent stem cells and upon the onset of differentiation Atoh8 was found to readily translocate into the nucleus raising several questions regarding its role in the exit of pluripotent state [14]. At the same time, its expression in human pluripotent stem cells is not evident. However, the current literature agrees with the fact that Atoh8 is activated following the onset of differentiation whether it is in mice- or human-derived pluripotent stem cells [20,21,22]. In addition, we previously showed that the loss of Atoh8 in murine derived pluripotent stem cells primes them towards mesendodermal fate by regulating genes involved in TGF-ß signaling [20].

Until now, the role of Atoh8 has been studied either in the context of pluripotency or murine organogenesis during development. However, its involvement in early embryonic morphogenesis following implantation, during gastrulation remained to be examined. This is particularly interesting because of the influence that TGF-ß and BMP signaling have on the early embryonic morphogenesis and at the same time on the expression of Atoh8. Recent studies performed to understand the transcriptional network involved in gastrulation at single-cell resolution confirmed the expression of Atoh8 in all three germ layers during gastrulation. However, in light of our recent report where we have shown that the absence of Atoh8 primes murine pluripotent stem cells towards mesendodermal fate, it would be interesting to know how Atoh8 is engaged in early embryonic development particularly during gastrulation [20,23,24].

#### 2.1.2. Atoh8 in Neurogenesis

During development, bHLH transcription factors regulate the expression of genes that are involved in cell fate determination and differentiation by acting as either transcriptional activators or repressors [25,26]. Atoh8 has been identified for the first time in the context of murine neurogenesis, it was shown to be expressed in the neuronal precursor cells of the ventricular zone and later in a subset of differentiating and mature neurons. Its misexpression in the developing retina was shown to induce neurogenesis while repressing gliogenesis. As it is expressed both in the neuronal precursor cells and later in the differentiating and mature neurons, it was implied that Atoh8 not only determines the fate of neuronal precursor cells but might also regulate the function of mature neurons [11]. In chicken, the homologue of Atoh8, Cath6 was found to be expressed in stem-cell-like progenitor cells in the marginal zone of the retina. Furthermore, this study characterized Cath6’s function as a transcription factor, the ‘Cath6’ protein was fused with the VP16 transactivation domain that could recruit the transcriptional machinery revealed that Cath6 could act as a transcriptional activator. However, the activation of Cath6 resulted in the inhibition of neuronal differentiation in the retina suggesting Cath6 as a negative regulator of neuronal differentiation that is unique to the Atonal family. Another study performed on mice also suggested Atoh8 as an important regulator of neuronal differentiation during retina development. Additionally, this study demonstrated that Atoh8 is activated by BMP14 which further acts as a modulator of retinal neuron differentiation. The loss of Atoh8 was further observed to inhibit the differentiation of the retinal stem cells [27].

#### 2.1.3. Atoh8 in Kidney Development

During kidney development, Atoh8 was primarily identified to be expressed in the metanephric mesenchyme and later in the tissues derived from it such as in the developing Bowman’s capsule and the tubule system with no evident expression in the uretic bud or uretic bud-derived cells. Intriguingly, the highest expression of Atoh8 was found in the least differentiated cell types of the nephrogenic zone with elevated levels of BMP4 signaling [28,29].

#### 2.1.4. Atoh8 in Pancreas Development

In 2008, Lynn and his colleagues identified Atoh8 expression in endocrine and exocrine precursor cells of the developing pancreas. This study emphasized Atoh8 as a direct target of Neurog3, which is essential for pancreatic development. It was further shown that Atoh8 possesses the ability to modulate the proendocrine functions of Neurog3 [30]. Similarly, another study performed on mouse embryonic stem cells also showed Atoh8 as a direct target of Neurog3 [31]. Lynn and colleagues further analyzed the mechanism of Atoh8 activation by Neurog3 and revealed the involved mechanism to be an epigenetic mechanism, where Neurog3 induces the conversion of a repressive bivalent chromatin state (H3K4me3 and H3K27me3) of Math6 to an active monovalent chromatin state (H3K4me4), which is a feature most commonly associated with the genes involved in the development where genes are poised for immediate activation by the bivalent repressive chromatin state [32]. Similarly, the analysis of genome-wide chromatin state in pluripotent stem cells also confirmed such a bivalent repressive state in the case of Atoh8 indicating the need for rapid activation of Atoh8 possibly for fine-tuning the process involved [33,34,35]. Altogether, these data show that Atoh8 is also controlled by epigenetic mechanisms.

Furthermore, in murine pancreatic ductal adenocarcinoma cells, it was shown that Atoh8 with the help of its proline-rich domain represses Neurog3-mediated gene function by competing with the transcriptional activity of E47 and Neurog3 [36]. As Atoh8 lacks repressor domains like the other bHLH proteins, it can be assumed that Atoh8 like the non-bHLH transcription factors acts as a repressor with the help of the proline-rich domain. Most recently, the same group has also generated conditional pancreatic Atoh8 knockout mice where they reported an increase in the δ-cell compartment of the pancreas in connection with a modest reduction in glucose and insulin levels [37].

#### 2.1.5. Atoh8 in Skeletal Muscle Development

During skeletal myogenesis, Atoh8 (Cath6) was identified to be predominantly expressed in the myotomal compartment of chicken embryos. To understand the significance of Atoh8 in myogenesis, Atoh8 expression was inhibited in the hypaxial myotome region, which resulted in the blockage of terminal differentiation of myoblasts restricting them to a pre-determined state. Similarly, experiments performed on the C2C12 murine myoblasts revealed a dynamic expression of Atoh8. Atoh8 was observed to upregulate following induction of differentiation suggesting a regulatory role during the transition of myoblasts from proliferative state to differentiation [38].

#### 2.1.6. Atoh8 in Heart Development

A study performed in order to understand the role of Atoh8 in the developing heart showed Atoh8 expression primarily in the atria. However, in mature animals, its expression was observed to be restricted to the right atrium suggesting a specific function. Apart from that, this study also identified a strong expression of Atoh8 in lung mesenchyme. Furthermore, subtle defects in the function of the lung, associated with mesenchymal-epithelial signaling, were also observed [39].

#### 2.1.7. Atoh8 in Cartilage and Bone Development

Atoh8 expression was first identified in the chondrocytes derived from proliferative and hypertrophic zones of the epiphysial growth plate [40]. Recently, a study performed by Schroeder and her colleagues has generated a conditional knockout model of Atoh8 specific to cartilage tissues. This study showed Atoh8 as a regulator of endochondral ossification by controlling the rate of proliferation and the onset of differentiation. The loss of Atoh8 during chondrogenesis resulted in the reduction of skeletal size [41]. Another study performed most recently on Zebrafish reiterated these findings revealing the expression of Atoh8 in the sclerotomes very early during development [42].

#### 2.1.8. Atoh8 in Placenta Development

Atoh8 was recently identified to play a major role in murine placenta development. Loss of Atoh8 was shown to lead to irregular development of the placenta during late mid-gestation with abnormal decidualization and vascularization, which ultimately led to hemorrhagic crisis and miscarriage of most of the fetuses with surprising survival of few fetuses despite their genotype, suggesting that Atoh8 has no or only a limited role to play during early embryonic development [43]. However, differentiation of pluripotent stem cells in vitro implied otherwise by priming the cells towards mesendodermal fate in the absence of Atoh8 implying its potential involvement in gastrulation [20]. One possible explanation regarding this difference could be that the loss of Atoh8 during gastrulation in vivo *or* in utero development is being compensated by either another bHLH factor as we observe in myogenesis or by another non-bHLH factor with functional similarity [44,45,46].

Previously, Atoh8 was shown to be a shear stress-responsive gene in human endothelial cells [47]. In addition to this, a recent study has indicated Atoh8 as a hypoxia-responsive gene that could attenuate the hypoxic response. Moreover, the same study also identified that Atoh8 knockout mice suffer from a condition similar to pulmonary arterial hypertension which is reported to have high morbidity and mortality in humans particularly during pregnancy [48,49]. Together with the fact that Atoh8 expression is restricted to the right atrium in adult mice, it can be hypothesized that the phenotype observed concerning placental development might be indeed because of pulmonary arterial hypertension which might not have been compensated by another bHLH factor or non-bHLH factor.

### 2.2. Atoh8 in Disease

#### 2.2.1. Atoh8 in Cancer

Given the ubiquitous expression and multiple regulatory roles of Atoh8 during embryonic development, it is not at all surprising to see its involvement in cancer and several other disorders. The high-grade gliomas are diagnosed on the basis of different molecular markers. The 1p19q codeletion is one characteristic marker for oligodendroglioma. In contrast, the epidermal growth factor receptor (EGFR) amplification is seen in glioblastoma. In 2008, a study performed to understand the two mutually exclusive groups of gliomas carrying ‘1p19q codeletion’ and ‘EGFR amplification’, identified Atoh8 as one of the multiple differentially regulated genes. Higher expression of Atoh8 was found to be correlated with the pro-neural group of gliomas particularly oligodendroglioma with ‘1p19q codeletion’ and lower expression of Atoh8 was found to be correlated with a proliferative and mesenchymal group of gliomas with ‘EGFR amplification’. Likewise, another study which was performed to ascertain the copy number variations in glioblastomas has also revealed aberrant expression of Atoh8 further suggesting it as a potential candidate involved in carcinogenesis [50,51]. Following this, another study which was investigating the effect of retinoic acid on glioblastoma stem cell-like cells also showed an increase in the expression of Atoh8 following treatment with retinoic acid which was found to be correlated with differentiation of the glioblastoma stem cell-like population [52]. In recent years, Atoh8 has been further studied in other types of cancers. In hepatitis B virus-associated hepatocellular carcinomas (HCC), reduced levels of Atoh8 were observed to be correlated with loss of tumor differentiation. Confirming the same, analysis of additional hepatic carcinoma cell lines such as HepG2, PLC8024 and CRL8064 also revealed a correlation between reduced Atoh8 expression and increased CD133 positive population. Additionally, the authors have also evaluated the effect of overexpression of Atoh8 in the hepatic carcinoma cell lines, which resulted in the reduction of the proliferation along with reduced invasive and migratory abilities of those cells. Lastly, overexpression of Atoh8 was also shown to reduce tumor formation and increase chemosensitivity in these cells. Most importantly, this study showed that Atoh8 could repress core regulators of pluripotency such as Oct4 and Nanog by binding to their E-Box sequences [22,53].

In nasopharyngeal carcinomas (NPC), similar to the findings in hepatic carcinomas, the inhibition of Atoh8 was shown to enhance the malignant phenotype, whereas its transgenic expression was shown to reverse the phenotype. In this study, the authors identified the silencing of Atoh8 in tumor cells as an epigenetic mechanism induced by the tumorigenic LMP1 (Latent Membrane Protein-1). In tumor cells, the occupancy of active H3K4me3 of the Atoh8 promoter sequence was found to be replaced by a repressive H3K27me3 mark. Lastly, this study further described Atoh8 as a regulator of epithelial-mesenchymal transition (EMT) because of its correlation with EMT markers [54].

In colorectal cancer (CRC), higher expression of Atoh8 was found to correlate with the poor prognosis of CRC patients. The knockdown of Atoh8 in CRC cells was shown to reduce proliferation with an accumulation of cells in the S phase of the cell cycle. Additionally, the depletion of Atoh8 in CRC cells was observed to increase apoptosis with no apparent changes to the migratory ability of the cells which is in clear contrast to the findings observed in HCC and NPC [55]. Another study, which used colorectal cancer cells as mimics of circulating tumor cells (m-CTCs), showed Atoh8 as a mechanosensor following their exposure to laminar shear stress. This study indicated that activation of Atoh8 in m-CTCs increased their plasticity and intravascular survival suggesting a promoting effect of Atoh8 [56].

In breast cancer, a study was performed to identify the enriched tissue-specific transcription factors which connected Atoh8 to the dysregulated transcriptional regulatory network which affected the proliferation, differentiation, cell adhesion and metastasis [57]. Interestingly, a most recent study has identified a novel isoform of Atoh8 called Atoh8-V1 in breast cancer, it was shown to be a negative prognostic marker with a high expression. Atoh8-V1 was further shown to bind and activate the RhoC promoter which in turn promotes metastasis in breast cancer. Similarly, downregulation of Atoh8-V1 was shown to correlate with reduced metastasis [13]. Given this new transcriptional variant, it would be interesting to see if the contradictory reports that are mentioned in the case of different cancers result from divergent functions of Atoh8 or because of the existence of more transcriptional variants.

In prostate cancer, Atoh8 was reported to be epigenetically active with high expression [58]. Similarly, in renal cell carcinoma, higher levels of Atoh8 was also shown as the worst prognostic marker [59].

#### 2.2.2. Atoh8 in Cellular Homeostasis

So far, Atoh8 has been implicated to be involved in cellular stress response, hypoxia response, and iron metabolism. Atoh8 was identified to be activated by shear stress during endothelial cell differentiation. It was further reported that overexpression of Atoh8 mimicked shear-stress treatment attributing a major role to Atoh8 in stress management. In addition to shear stress, IFN-γ, TNF-α and oxidative stress were shown to act upstream and activate Atoh8 expression in endothelial cells. Furthermore, eNOS which plays a major role in endothelial cell protection, nitric oxide production was shown to be positively regulated, at the same time as a direct target of Atoh8 [21,47]. The phenotype observed in Atoh8 knockout mice which was similar to pulmonary arterial hypertension together with the reduced levels of the eNOS following knock-down or absence of Atoh8 correlates with the phenotype of defective placental development observed in Atoh8 knockout mice (Table 1) [21,43,48]. In addition to this, it would also be of great interest to check if Atoh8-associated stress response is restricted to the endothelial cells or if it could perform a similar function in other cell types as it is ubiquitously expressed.

Atoh8 was identified as a downstream factor for activating transcription factor 4 (ATF4) in pancreatic ß-cells. ATF4 is a transcriptional regulator for integrated stress response (ISR) and unfolded proteins. Severe diabetes is led by dysregulation of ISR. Thereby ATF4 senses endoplasmic reticulum stress in cellular pathologies and is responsible for the maintenance of homeostasis in the endoplasmic reticulum. The ISR enhancer Sephin1 increased the ATF4 expression in the pancreatic islets and increased the insulin secretion [61].

Lately, Atoh8 was also shown to regulate hypoxic response. A study by Morikawa’s group showed that Atoh8 binds hypoxia-inducible factor 2α (HIF-2α) and decreases its abundance leading to the attenuation of the hypoxic response. It was further shown that the Alk1/Smad/Atoh8 axis acts as a regulator of the hypoxic response. Interestingly, analysis performed on HPAECs showed the HLH domain of Atoh8 to be crucial for hypoxic response with no functional role of the basic domain [48]. Recently, another study performed on hypoxia adaptation of Tibetan pigs also showed Atoh8 as a potential regulator of hypoxia, which has the potential to modulate TGF-ß and PI3K-AKT signaling [62].

**Figure 3:**
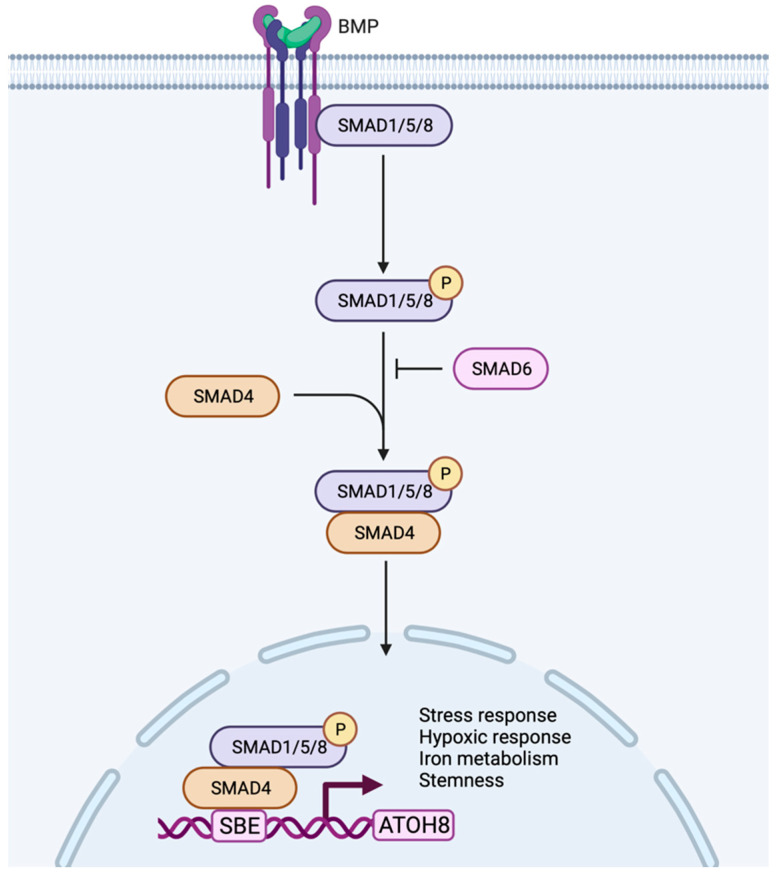
Working model of Atoh8 activation by canonical BMP signaling pathway. In the canonical pathway, BMPs transduce their signal by assembling type I or type II receptors to form a hetero-tetrameric complex. The assembly of receptors initiates transphosphorylation of type I receptors by type II receptors which further phosphorylates and activates R-Smads (Smad 1/5/8). The activated Smad1/5/8 interacts with Co-Smad (Smad4) to form a complex that further translocate into the nucleus to bind to the Smad Binding Element (SBE) of Atoh8 to initiate its transcription. Based on the current literature, the activated Atoh8 can be considered to regulate stress response, hypoxic response, iron metabolism and stemness of the cells.

In addition to stress and hypoxia regulation, Atoh8 was also shown to regulate metabolism, especially iron metabolism. Atoh8 was observed to regulate hepcidin which prevents the overloading of iron in the body. In hepatocytes, BMP6 was shown to activate Atoh8 (Figure 3), which further binds to the promoter of hepcidin and promotes hepcidin production, ultimately regulating the uptake of iron by the duodenal epithelium [63,64]. Despite the existence of multiple Atoh8 knockout mouse models, no phenotype concerning iron metabolism or liver has been reported so far.

Intriguingly, a recent study showed an increase in the number of M-Cells in the intestine of Atoh8 knockout mice whose main function is to uptake and transfer the bacterial antigens to dendritic cells as part of immunosurveillance. Iron plays a crucial role in bacterial growth, overloading of iron leads to a significant increase in gut microbiota resulting in inflammation. It is logical to think that the increase in the number of M-Cells in the case of Atoh8 knockout mice could be a compensatory mechanism adapted by these animals to counter prolonged bacterial infections [65,66].

## 3. Conclusions and Future Perspective

Atoh8 is a transcription factor that belongs to a large superfamily of transcriptional regulators called bHLH proteins. In spite of two decades of research, multiple questions regarding its molecular function and involved mechanisms remain elusive. The current literature shows that Atoh8 is involved in the regulation of pluripotency and organogenesis during embryonic development. Additionally, Atoh8 was also shown to regulate several physiological processes such as stress response, hypoxic response and iron homeostasis in adult and mature animals. It has further been attributed to influence cellular mechanisms such as proliferation, cellular survival, EMT in cancers and MET in reprogramming.

So far, multiple studies performed in different contexts confirmed that Atoh8 is a direct target of the Smad-dependent TGF-ß/BMP signaling pathway. Although Atoh8 seems to be a tissue-restricted factor during organogenesis controlling cellular fate and differentiation, its ubiquitous expression in adult animals suggests a much larger role. Based on contemporary studies, Atoh8 can be considered as a negative regulator of multiple basic cellular mechanisms starting from proliferation to metabolism. Since these mechanisms are inextricably connected to proper cellular function, Atoh8 must be thoroughly studied in the regulation of basic cellular mechanisms such as cell metabolism, cell cycle, apoptosis and autophagy.

## Figures and Tables

**Table 1:** List of Atoh8 mutant mice and observed phenotypes.

Deletion of Genomic Region	Reported Phenotype	Mouse Strain	References
Exon 1 and 2	Early embryonic lethality	C57BL/6	[30]
Exon 1	Normal	Mixed background C57BL/6 and SV/129	[39]
Exon 1	Defective placenta development	C57BL/6NJ	[43]
Exon 1	Pulmonary arterial hypertension and delayed retinal angiogenesis	C57BL/6	[48]
Exon 1	Hearing loss	Mixed background C57BL/6J and 129S6	[60]

## Data Availability

Not applicable.
